# Commentary: Lower hydration status increased diabetic retinopathy among middle-aged adults and older adults: results from NHANES 2005–2008

**DOI:** 10.3389/fpubh.2024.1354496

**Published:** 2024-03-13

**Authors:** Xiao Li, Zhihui Lu, Meirong Chen

**Affiliations:** ^1^First Clinical Medical College, Shandong University of Traditional Chinese Medicine, Jinan, China; ^2^Department of Ophthalmology, Shandong Hospital of Traditional Chinese Medicine, Jinan, China

**Keywords:** NHANES, hydration status, diabetic retinopathy, cross-sectional studies, osmotic pressure

## Introduction

We read with interest the publication “*Lower hydration status increased diabetic retinopathy among middle-aged adults and older adults: results from NHANES 2005–2008*” by Zhang et al. (1). The authors used National Health and Nutrition Examination Survey (NHANES) 2005**–**2008 to explore the association between hydration status and diabetic retinopathy (DR) in middle-aged and older adults in the United States. In this study, the authors assessed patient hydration status by serum osmolality. The study found that patients in the highest quartile of serum osmolality had a higher risk of developing DR. They also categorized DR by severity and further concluded that adults with low hydration status were at higher risk of developing moderate/severe non-proliferative retinopathy and proliferative DR. This is a very interesting study that, for the first time, investigates the association between hydration status and DR. We highly appreciate the study conducted by the authors and support their conclusions. However, we have some questions about the process of this study.

## Is the inclusion process correct?

In this study, the authors selected 5,704 subjects who participated in retinal imaging examinations in NHANES 2005–2008 and then excluded subjects who lacked a range of data on education, body mass index (BMI), drinking status, smoking status, and so on, totaling 484. A total of 5,220 people ultimately participated in the study. Subjects were then categorized according to whether they had DR or not, with a total of 641 in the DR group and 4,579 in the non-DR group. The association between hydration status and DR was then explored using multivariate logistic regression modeling and further analyzed according to different severities of DR and different age segments (mainly for people aged 65 years and older). It was concluded that there was a positive association between hydration status and DR. The study design was very detailed and comprehensive, but we believe it may not be accurate. Because the focus of this study was to explore the association between hydration status and DR in middle-aged and older Americans, we felt that this study should have first excluded non-diabetic patients. According to the retinal imaging program provided by NHANES, all participants aged 40 years and older who did not meet any of the exclusion criteria were eligible for the study (2). This means that subjects could participate in retinal imaging regardless of whether they had diabetes. However, in this study, the authors did not mention whether subjects without diabetes were excluded or how diabetes was defined. We utilized the NHANES questionnaire with information about diabetes, i.e., “Have you been told by your physician that you have diabetes?” and found that only 884 patients were diagnosed with diabetes in NHANES 2005–2008, which is a significant difference from the 5,220 patients included in the study. We believe that this is very important and may have influenced the results of the study.

## Discussion

This study was innovative in exploring the association between hydration status and DR in middle-aged and older adults in the United States. However, we hope that the authors would have paid attention to the issues we mentioned so that readers could draw more accurate conclusions from the study.

## Author contributions

XL: Writing – original draft. ZL: Writing – original draft. MC: Writing – review & editing.
